# The Effect of an Educational Intervention Based on the Health Action Process Approach on Nurses’ Communication Skills

**DOI:** 10.17533/udea.iee.v42n1e13

**Published:** 2024-04-29

**Authors:** Mojtaba Fattahi Ardakani, Ahmad Sotoudeh, Ali Asadian, Sara Heydari, Moradali Zareipour

**Affiliations:** 1 . Continuous Education, Shahid Sadoughi University of Medical Sciences, Yazd, Iran. Email: mjfattahi57@gmail.com Shahid Sadoughi Shahid Sadoughi University of Medical Sciences Yazd Iran mjfattahi57@gmail.com; 2 .Department of Public Health, Bushehr University of Medical Sciences, Bushehr, Iran. Email: sotoudeh_ahmad@yahoo.com Bushehr University of Medical Sciences Department of Public Health Bushehr University of Medical Sciences Bushehr Iran sotoudeh_ahmad@yahoo.com; 3 .Social Determinants in Health Promotion Research Center, Hormozgan University of Medical Sciences, Bandar Abbas, Iran. Email: a.asadian.a@gmail.com Hormozgan University of Medical Sciences Social Determinants in Health Promotion Research Center Hormozgan University of Medical Sciences Bandar Abbas Iran a.asadian.a@gmail.com; 4 .Assistant Professor of Medical Education, Shahid Sadoughi University of Medical Sciences, Yazd, Iran. Email: s.heydari.287@gmail.com Shahid Sadoughi Shahid Sadoughi University of Medical Sciences Yazd Iran s.heydari.287@gmail.com; 5 .Department of public Health, Khoy University of Medical Sciences, Khoy, Iran. Email: z.morad@yahoo.com Corresponding Author Khoy University of Medical Sciences Department of public Health Khoy University of Medical Sciences Khoy Iran z.morad@yahoo.com

**Keywords:** self-efficacy, nurse, health action process approach, communication, education intervention., autoeficacia, enfermera, enfoque del proceso de acción sanitaria, comunicación, intervención educativa., autoeficácia, enfermeiro, abordagem do processo de ação em saúde, comunicação, intervenção educacional.

## Abstract

**Objective.:**

This study aimed to the effects of the Health Action Process Approach (HAPA) in promoting the quality of nurses' communication skills among nurses.

**Methods.:**

The present quasi-experimental research was conducted on 148 nurses (76 in the intervention and 72 in the control group) in Yazd province (Iran). In this study, the total number of nurses in one hospital was selected as the intervention group, while the nurses from another hospital were chosen as the control group. The participants were recruited from public hospitals in Ardakan and Meibod cities. The data collection instrument was a questionnaire based on the Health Action Process Approach (HAPA) Constructs and a communicative skill questionnaire. The data were collected from the two groups before, one month after, and four months after the intervention. The control group did not receive any educational training during the course of the study.

**Results.:**

In the pretest, no statistically significant difference was found between the intervention and control groups regarding the behavioral stages of effective communication with patients. In the posttest, the mean task self-efficacy score was significantly increased in the intervention group compared to the control (*p*<0.001). The mean coping self-efficacy score was also significantly higher in the intervention group than the control in the posttest (*p*<0.001). Moreover, the mean coping planning score was significantly increased in the post-test intervention group(*p*<0.001). The mean communicative skill score was also significantly increased in the intervention group compared to the post-test control (*p*=0.03).

**Conclusion.:**

The intervention used in the present study based on the target model (HAPA) significantly affected nurses’ self-efficacy and communicative skills in the experimental group.

## Resumen

**Objetivo.:**

Evaluar el efecto del enfoque del proceso de acción sanitaria (Health Action Process Approach (HAPA), en inglés) en la promoción de la calidad de las habilidades de comunicación de las enfermeras.

**Métodos.:**

La presente investigación cuasiexperimental se llevó a cabo con 148 enfermeras (76 en el grupo de intervención y 72 en el de control) de la provincia de Yazd (Irán). Los participantes fueron reclutados en los hospitales públicos de las ciudades de Ardakan y Meibod. El instrumento de recogida de datos fue un cuestionario basado en los constructos HAPA y un cuestionario de habilidades comunicativas. Se recogieron datos de los dos grupos antes, un mes después y cuatro meses después de la intervención. El grupo de control no recibió ninguna formación educativa durante el estudio.

**Resultados.:**

En la preprueba, no se encontraron diferencias estadísticamente significativas entre los grupos de intervención y de control en cuanto a las etapas conductuales de la comunicación eficaz con los pacientes. En la prueba posterior, la puntuación media de autoeficacia en la tarea aumentó significativamente en el grupo de intervención en comparación con el grupo de control (*p* **<**0.001). La puntuación media de autoeficacia en el afrontamiento también fue significativamente mayor en el grupo de intervención que en el grupo de control en el postest (*p* **<**0.001). Además, la puntuación media en planificación del afrontamiento aumentó significativamente en el grupo de intervención después de la prueba (*p* **<**0.001). La puntuación media en habilidades comunicativas también aumentó significativamente en el grupo de intervención en comparación con el grupo de control después de la prueba (*p* **=**0.03).

**Conclusión.:**

La intervención utilizada en el presente estudio basada en el modelo HAPA mejoró significativamente la autoeficacia y las habilidades comunicativas de las enfermeras del grupo experimental.

## Resumo

**Objetivo.:**

Avaliar o efeito da Abordagem do Processo de Ação em Saúde (HAPA) na promoção da qualidade das habilidades de comunicação dos enfermeiros.

**Métodos.:**

A presente pesquisa quase-experimental foi realizada com 148 enfermeiros (76 no grupo de intervenção e 72 no grupo de controle) da província de Yazd (Irã). Os participantes foram recrutados em hospitais públicos nas cidades de Ardakan e Meibod. O instrumento de coleta de dados foi um questionário baseado nos construtos do HAPA e um questionário de habilidades de comunicação. Os dados foram coletados dos dois grupos antes, um mês depois e quatro meses após a intervenção. O grupo de controle não recebeu nenhum treinamento educacional durante o estudo.

**Resultados.:**

No pré-teste, não foram encontradas diferenças estatisticamente significativas entre os grupos de intervenção e controle em termos de estágios comportamentais da comunicação eficaz com os pacientes. No pós-teste, a pontuação média de autoeficácia na tarefa aumentou significativamente no grupo de intervenção em comparação com o grupo de controle (*p*<0.001). A pontuação média de autoeficácia de enfrentamento também foi significativamente maior no grupo de intervenção do que no grupo de controle no pós-teste **(** *p*<0.001). Além disso, a pontuação média do planejamento de enfrentamento aumentou significativamente no grupo de intervenção após o pós-teste (*p*<0.001). A pontuação média em habilidades de comunicação também aumentou significativamente no grupo de intervenção em comparação com o grupo de controle no pós-teste (*p*=0.03).

**Conclusão.:**

A intervenção usada no presente estudo com base no modelo HAPA melhorou significativamente a autoeficácia e as habilidades de comunicação dos enfermeiros do grupo experimental.

## Introduction

Nurse-patient relations are at the core of assessing the quality of nursing care provision. Besides care services, patients need empathy, understanding of personal needs, clarity of the treatment objective, awareness of the trend of the disease, and lower anxiety. These needs can be met if effective communication is facilitated.^1^^,^^2^ Moreover, through effective nurse-patient communication, the patient trusts the therapeutic measures and adheres more to medical advice. If a nurse is very well aware of a patient's needs and feels this warmth and friendliness in communicating with the nurse, she/he can enjoy his/her stay in the hospital. This experience will, later on, affect the choice of the hospital to visit.^2^ Despite the seemingly evident significance of nurse-patient relations, several studies reported a high rate of patient complaints about these relations.^3^^,^^4^

Similarly, different studies in Iran reported complaints about the quality of care and how the medical staff treats patients.^3^^,^^5^^,^^6^ Universities offer courses to teach how to communicate effectively with patients. However, these instructions do not always translate into clinical attempts. A body of research found the prevalence of ineffective nurse-patient communication. Among the underlying reasons can be the nurses' underestimation of the relations, unawareness of the trend of disease, unwillingness to communicate, low self-confidence, and lack of communicative knowledge and skills.^(6, 7)^ Promoting communication skills requires a well-organized plan. Communication skill is acquisitional, similar to many other skills, and can be promoted via a comprehensive educational program and strategies to reduce communication barriers.^8^ Using a theoretical framework or model can be an interventional strategy to achieve this goal. Using a framework or an approach acts as a map to achieve a goal.^9^

The Health Action Process Approach (HAPA) is a socio-cognitive model and a psychological framework in health education. This model was employed in the present study.^10^ In this model, the behavior change process involves two motivational and a volitional phases. The former involves an individual's Intention to act in a certain way or change a high-risk behavior. The latter involves the Intention being turned into actual behavior and involves three further stages, initiation, maintenance, and recovery.^10^ In the first motivational stage, an individual intends to do something. In this stage, the perceived threat is considered a primary precursor.^10^ In the motivational stage, when the individual strikes a balance between the benefits and barriers of a certain behavior, expecting positive outcomes is always important. Moreover, to act as desired, she/he needs to fully believe in his/her capabilities, which is known as perceived self-efficacy. Perceived self-efficacy is positively correlated with the expected positive outcomes. Both are involved in forming Intention. Once the individual is willing to show a certain healthy behavior, Intention is changed into detailed instructions. Therefore, the next stage should be analyzed into planning for action, coping, self-efficacy, and recovery.^10^^,^^11^ There is research evidence that if someone aims to perpetuate a behavior, s/he needs appropriate planning and strategies to maintain the new behavior. 

Many people intend to adopt a new behavior but quit it together for lack of the required planning. They, thus, easily turn back to their old behavior.^12^ Considering the constructs within this approach, before promoting a certain behavior or quitting an old behavior, one needs to have a high perceived threat, positive outcome expectancy, and high self-efficacy. Once the new behavior is initiated, it is of utmost importance to maintain it. Thus, planning to maintain the target behavior is a key factor. The volitional phase of the HAPA requires interventions to increase coping self-efficacy, behavior planning, and coping planning.^13^^,^^14^ A key property of this approach with HAPA is attention to the barriers to behavior planning and coping planning. The findings reported by Bodys-Cupak et al. showed that nursing students who had better planning and strategy use to manage their work challenges could better manage their stress during the training course.^13^ In their research, Parle et al. used the outcome expectancy, self-efficacy, and self-regulation strategies to promote communication skills, which led to the doctors' and nurses' improved communication skills and self-regulation strategies.^15^A review of the interventional studies promoting nurses' communication skills showed that the focus in most studies had been more on the motivational aspect of the tendency toward a behavior change, and no regular plan has been made to promote communication skills.^16^^-^^19^

What seems to be lacking is a program based on a planned approach to remove barriers to communication. Many people tend to change their behavior. After a short while, unfortunately, due to the lack of planning, the new behavior does not persist, and the behavior change occurs just for a short time. Therefore, like other behavior change strategies, promoting communication skills requires planning.^12^ As the HAPA consists of two phases, motivational and volitional, it can be used as an effective strategy for promoting communication skills. Therefore, this study aimed to enhance nurses' communication skills through an educational intervention based on the Health Action Process Approach.

## Methods

### Research Design and Participants

In the present quasi-experimental research, 148 nurses were selected to work in general wards [medical, Surgery, cardiac, women, and children] of two public hospitals in Ardakan and Meibod in Yazd province, Iran, in 2021. As some nurses from one hospital worked in multiple departments, randomization was not feasible, and the potential for shared educational interventions could impact the outcomes. Therefore, nurses from one hospital were selected randomly as the intervention group, and nurses from another hospital were chosen as the control group. Additionally, in terms of culture, education, and other demographic variables, there were no significant differences due to the proximity of the two hospitals. The intervention group received educational interventions based on HAPA, while no educational intervention was considered for the control group. The inclusion criteria were holding a diploma or higher and a nursing experience of at least six months in hospitals. The exclusion criteria were failing to respond to all questions or working part-time in the hospital. Besides, the participants were supposed to stay in the hospital for at least six months after questionnaire completion. Therefore, nurses who were prone to work force redundancy or termination of work, those working on a mission, and those who were pregnant and approaching childbirth were excluded from the study. All nurses who met the inclusion criteria were invited to participate in the study. 

To prevent education bias and its effect on the control group, nurses from one hospital were assigned to the intervention group and those from the other hospital to the control group. Therefore, one hospital was randomly selected for the intervention and the other for the control. The randomization was performed using a coin flip, resulting in one hospital being selected as the intervention group and another as the control group. The sample size in each group was estimated at 61 with an attrition rate of 20% [n=13], an estimated error of α=0.05 and β=0.2. It was estimated at 74 using the following formula. Therefore, a total number of 148 was finally set. 




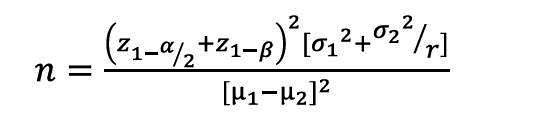




**Figure 1:**
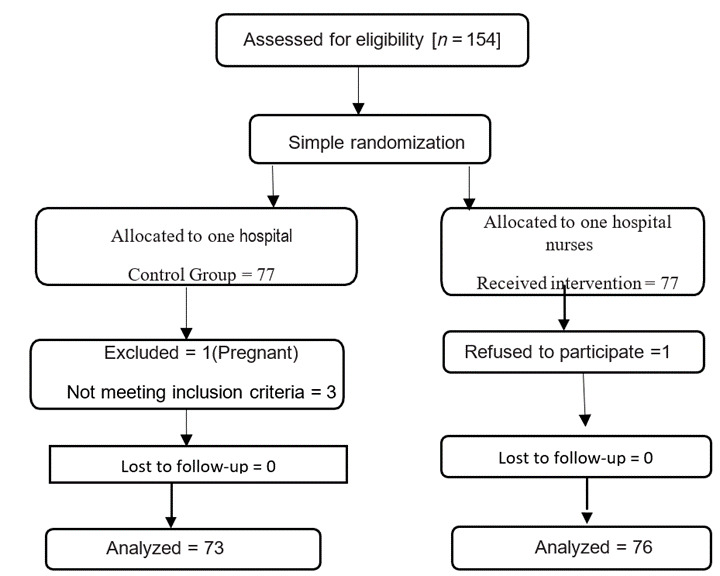
The flow diagram of the study

### Data Collection and Instrumentation

The data collection instrument was comprised of three parts: (i) The participants' demographic information includes their age, sex, education, ward of affiliation, and work experience; (ii) The self-rating communication skills questionnaire with 26 items to measure a respondent's communication skills. The responses were scored between 0 and 100. The minimum score for communication skills was 0, and the maximum score was 2,600. The reliability and validity of the questionnaire were already substantiated in the previous study.^20^

The communication skills questionnaire based on HAPA: The researcher-made HAPA questionnaire was developed based on the perceived threat, outcome expectancies, task self-efficacy, Intention, behavior planning, coping self-efficacy, coping planning constructs. Each scale consisted of a stem and several items. The respondents were asked to read the branch and answer the items on a 5-point Likert scale. The CVI (0.89) and CVR (0.79) were estimated to test the content validity. To this aim, eight health education and promotion experts were consulted to comment on the item's simplicity, relevance, and clarity. The instrument was also checked for internal consistency, using Cronbach's alpha. This value was estimated above .7, and the results can be seen in the study published by Ardakani *et al*.^21^

### Intervention

Before the intervention, the control and intervention group nurses both responded to the communication skill and HAPA questionnaires. Then, the intervention began in two phases intending to promote communication skills: 

*(i) The motivational phrase*. The education aiming to increase Motivation for promoting communication skills was implemented in this phase. During these sessions, comprehensive educational methods were used, such as lectures, group discussion, sharing of communication experiences in the presence of nurses who had successfully communicated with patients. This phase took 2-hour sessions held in 4 weeks. Meanwhile, these sessions were not fit for the control group for any teaching of communication skills in the hospital. Immediately after the educational intervention in the motivational phase, part of the questionnaire developed based on the HAPA, including the motivational stage [task self-efficacy, Intention, outcome expectancy, and perceived threat] was provided for nurses in the intervention and control groups.

*(ii)The volitional phase*. In this phase, nurses in the intervention group were selected based on the results of the motivational phase to enter the volitional phase. To this aim, again, all nurses in the intervention group were invited to take part in 3 more sessions, each taking 2 hours long. In these additional sessions, the principles of communication skills were taught according to the constructs of the volitional phase. The educational content addressed the barriers to verbal and nonverbal communication and the basics of effective nurse-patient communication. After the educational intervention, the nurses were asked to record communicating with patients using an evaluation checklist. Nurses were supposed to rate their level of communicating with patients, such as effective listening to patients, empathy, verbal communication, and nonverbal communication, by assigning a score to each. They were also asked to describe their spirits, thoughts, and feelings while communicating with patients. 

This self-rating evaluation checklist was developed based on a book on the essential communication skills for nurses^22^ and the reflection model.^23^ Nurses evaluated themselves by recording their thoughts, feelings, and insights. They got aware of their positive and negative perceptions and attitudes. This awareness helped them reinforce positive emotions and reduce the negative. If it was hard for them to write down the details, an Android application was also developed to be easily installed on mobile phone devices to be used daily for 30 days. Also, head nurses held within-ward sessions on how to implement the program to support communication skills adequately. Again, one month after the classes, all nurses (in the intervention group and controls] were asked to respond to the questionnaire developed based on the volitional phase [coping self-efficacy, behavior planning, and coping planning]. Four months after the intervention, all control and intervention groups participants were asked to complete a questionnaire comprised of the coping self-efficacy, coping planning constructs, and the communication skills questionnaire.

### Data analysis

To statistically analyze the data, SPSS18 was used. Paired-samples and independent-samples T-tests were run to test the within- and between-group differences in the scores of HAPA constructs and communication skills. Besides, one-way ANOVA was used with repeated measures to compare the coping self-efficacy and coping planning scores in three points of time at the p<.05 significance level. 

### Ethical Considerations

The participants were ensured of the confidentiality of the information they provided. Then, they were provided with questionnaires to fill out. Participation in the study was voluntary. The ethical committee approved the Yazd University of medical sciences [#IRSSU.SPH.REC.1395.76].

## Results

Among the 148 participants in this research, 76 were assigned to the intervention and 72 to the control group. The mean age of the nurses was 33.88±7.24 years (R=22-52). Their mean length of work experience was 10.57±8 years (R=1-27). No statistically significant difference was found between the two groups before the intervention in terms of age, sex, work experience and education. (Table 1)

**Table 1:** Socio-demographic distribution by study groups

Variables	Categories	Intervention group *n* (%)	Control group *n* (%)	Total *n* (%)	(2	*p*-value
Sex	Male	9 (6.1)	5 (3.4)	15 (9.5)	1.	0.2
	Female	67 (45.3)	67 (45.3)	134 (90.5)	03	2
Education	Diploma	8 (10.5)	5 (6.9)	13 (8.8)		
	Associate degree	4 (5.3)	3 (4.2)	7 (14.7)	0.94	0.87
	Bachelor's degree	60 (78.9)	59 (81.9)	119 (80.4)		
	Master's degree	4 (5.3)	5 (6.9)	9 (6.1)		

The results indicated in Table 2 show the difference between the research groups in the behavioral stages before the intervention in the pretest. Overall, in both groups, 6.8% belonged to the pre-intention, 19.6% to the Intention, and 75.9% to the behavioral stages. Moreover, according to Table 3, no one from the intervention group was in the pre-intention stage after the motivational intervention. The nurses' affiliation with the behavioral stages was significantly different from the control group (*p*<0.001). As shown in the Table 3, the mean score of perceived threat was significantly higher in the control group before the intervention (*p*=0.05). Also, the motivational intervention led to no significant increase in the outcome expectancy in the intervention group (*p*=0.21). However, the increased mean difference in this group was statistically significant compared to the control group (*p*=0.06). The mean scores of behavioral Intention, task self-efficacy, and behavior planning in the two groups were not significantly different before the intervention, after the motivational intervention and the planning intervention, the mean scores of behavioral Intention (*p*<0.001), task self-efficacy (*p*<0.001) and behavior planning (*p*<0.001) showed a statistically significant increase in the intervention group compared to the control

**Table 2:** Between-group comparison of effective communication skills based on chi-squared test results before the intervention

*p*-value	(2		Total		Control group		Intervention group	Behavioral Stages of Effective Nurse-patient Communication Before Intervention
		%	*n*	%	*n*	%	*n*	
0.77	1.12	6.8	10	6.9	5	6.6	5	No intention for effective communication
		19.6	29	20.8	15	18.4	14	They were eager to learn effective communication skills (intention stage)
		30.4	45	33.3	25	27.6	21	They attempted to communicate effectively but found it hard.
		43.2	64	38.9	28	47.4	36	It was easy for them to communicate effectively.

**Table 3:** Between-group comparison of the model constructs before and immediately after the motivational phase

Independent-samples T-test	Mean±SD			Variables
	Control group	Intervention group		
t=-1.97, *p*=0.05	5.75±55.87	8.07±53.78	Before intervention	Perceived threat
t=-0.41, *p*=0.68	8.54±57.69	5.75±55.87	After intervention	
t=1.34, *p*=0.18	1.61±9.27 1.47 0.14	3.39±6.74 4.38 <0.001	Mean difference Student t test *p*-value	
t=-1.66, *p*=0.1	3.37±37.52	3.37±36.36	Before Intervention	Outcome expectancies
t=0.62, *p*=0.54	4.31±36.52	3.14±36.88	After intervention	
t=1.85, *p*=0.06	-0.81±4.95 1.40 0.16	0.394±2.74 1.25 0.21	Mean difference Student t test *p*-value	
t=-1.77, *p*=0.09	3.42±27.45	4.14±26.38	Before Intervention	Task self-efficacy
t=3.26, *p*<0.001	4.75±27.47	3.01±29.06	After intervention	
t=4.59, *p*<0.001	0.013±4.72 0.02 0.98	3.22±4.14 7.67 0.001	Mean difference Student t test *p*-value	
t=0.74, *p*=0.46	3.04±0.94	3.15±0.95	Before intervention	Intention
t=3.56, *p*<0.001	2.98±0.98	3.46±0.59	After intervention	
t=2.46, *p*=0.015	0.05±0.42- 0.42 0.67	0.3±0.61 4.31 0.001	Mean difference Student t-test *p*-value

According to the findings summarized in Table 4, the mean score of communication skills was significantly increased in the intervention group after the intervention compared to the control (*p*=0.03).

**Table 4:** Comparison of communication skills between groups before and four months after the intervention

Mean±SD			
Independent-sample t-test	Control group	Intervention group	
t=-0.52, *p*=0.60	2204.98±205.88	2185.13±251.06	Before Intervention
t=2.14, *p*=0.03	2210.26±219.06	2287.57±220.26	After intervention
t=2.67, *p*=0.008	5.27±190.68 0.18 0.85	102.44±190.68 4.68 0.001	Mean difference Student t-test *p*-value

The mean score of coping self-efficacy in the intervention group one and four months after the intervention was significantly increased. The effect size was high (*p*<0.001). This increased difference was not observed in the mean score of the control group (*p*=0.64). The intervention in the volitional phase and stress management programs led to an increase in the mean coping self-efficacy score among nurses in the intervention group. The increased mean score of coping planning was a statistically significant one and four months after the intervention (*p*<0.001). This difference between the mean scores of nurses in the control group was not statistically significant (*p*=0.95) (Table 5).

**Table 5:** Between-group comparison of the coping score before, 1 and 4 months after the intervention

Variables		Before Intervention Mean±SD	1 month after Intervention Mean±SD	4 months after intervention Mean±SD	F	*p*-value	Partial Eta squared
Coping self-efficacy	Intervention	29.05±5.11	32.96±3.86	33.40±3.58	44.34	0.001	0.54
	Control	30.22±4.1	30.88±4.36	30.98±3.66	0.81	0.64	0.01
	*p*-value*	0.13	0.003	0.001			
Coping planning	Intervention	24.09±5.36	27.57±3.03	27.82±2.97	25.93	0.001	0.41
	Control	25.75±5.06	25.55±4.46	25.54±4.17	0.48	0.95	0.001
		*p*-value*		p=0.13	p=0.003		p<0.18				

* Independent-samples t-test *p*-value

## Discussion

This study aimed to enhance the communication skills of nurses using HAPA. The results revealed that education intervention led individuals in the intervention group to progress from the pre-intentional stage to the intention stage significantly more than the control group. 

 Moreover, the number of people in the intervention group who found it easy to communicate was significantly increased compared to the control group. The present findings prove the effectiveness of the intervention in improving nurses' willingness to acquire communication skills and promote communication skills. No such difference was found within the control group. Thus, it can be concluded that the motivational intervention managed to increase nurses' tendency to learn communication skills. 

Many studies have been conducted so far based on the HAPA. However, in none of them, the phase to which the participants belong is mentioned. Yet, in their study, Joveini *et al*.^24^ compared intenders and non-intenders. In this research, hookah smokers took part in an educational intervention in which the Motivation for ceasing hookahs and the perceived threat of the habit were investigated. The results confirmed the HAPA constructs, and after the educational intervention, the students in the intervention group showed a higher tendency to cease hookah smoking. These interventions were all based on the significance of communication skills in lowering the risks of ineffective communication. These include no diagnosis of background diseases, delayed diagnosis of a medical condition, and complaints about the quality-of-care services. As investigated and reported by the patient security committee, the main reason for 66% of medical errors is the lack of effective nurse-patient communication.^24^ Previous studies explored the correlation between perceived threat and health-protective behaviors and showed that a higher perceived threat is accompanied by more participation in showing health-related protective behaviors.^25^ Those who better communicate with patients are more susceptible to the threat of ineffective communication.^26^^,^^27^ According to Schwartz, the minimum perceived threat level should exist in people who have not yet intended to communicate effectively.^12^ In the present research, statistically significant differences were found in perceived threat before and after the intervention in the intervention group. This difference shows the effectiveness of the educational intervention in keeping nurses aware of the significance of effective communication. As also emphasized in the HAPA, the perceived threat is a construct that, if increased, can push people towards risk avoidance behaviors or promotive behaviors. In the research work by Lhakhang *et al*.,^28^ contrary to the present study, the intervention did not manage to increase perceived threat because decision-making interventions were used rather than promotive interventions to affect perceived threat. In their research, Zhang *et al*. reported a significantly higher perceived threat score in the intervention group [than the control], consistent with the present finding.^29^

In the present research, participants in both groups had a high outcome expectancy score. After the intervention, no statistically significant difference was found between the outcome expectancy score of the two groups. Thus, the intervention did not increase the mean score of the participants' outcome expectancy. No increase in the mean outcome expectancy score can be due to the high outcome expectancy before the intervention. Before the intervention, 90% of the maximum score was gained. Nurses believed that showing effective communicative skills could improve their life and work objectives. Liu et al. aimed to explore the effect of an educational intervention for communicative skills in nurses in charge of patients with cancer. They observed a significant increase in the outcome expectancy of the intervention group. The nurses gained 73% of the maximum outcome expectancy score.^30^ In the present study, participants in both groups had a high task self-efficacy score. After the intervention and teaching communication skills, the mean score of task self-efficacy was significantly increased in the intervention group. This finding indicates the increased level of nurses' self-belief in communicating effectively. Some other studies employed different modes of educational intervention such as role-play, lecture, and self-rated communication skills. They managed to increase nurses' self-efficacy in communication skills after the intervention compared to before the intervention. This is consistent with the present finding.^17^^,^^31^

The ineffective belief communication can be increased by teaching the key factors involved and identifying barriers to effective communication such as different age groups, sexes, cultures, and shared experiences of lacking effective communication with patients. In a study conducted by Owen and Rowbotham, which aimed to explore the effect of education through simulation, nurses showed higher communication self-efficacy after the educational intervention than the control group. ^32^ As pinpointed by Bong, teaching specific skills, representations, and role-plays can help improve people's skills and self-efficacy in showing the target behavior.^33^

In the present research, the mean score of behavioral Intention was significantly increased in the intervention group than in the control group. Similarly, Chiang *Et al*. investigated an increase in the physical activity of patients with spinal injury and realized that the intervention managed to increase behavioral Intention in the intervention group.^34^ This is consistent with the present research. The findings summarized in Table 5 showed that the mean coping self-efficacy score was significantly higher in the intervention group than the control in two-time points [1 and 4 months after the intervention]. Interventional measures can help promote coping self-efficacy through planning to cope with barriers, create perceived capability and emphasize problems such as hard work, business with work, and willingness to return to old habits or behaviors. 

In the present research, no statistically significant difference was found between the two groups in the mean score of planning for action before the intervention. However, after the educational intervention, the mean score of action planning was significantly higher in the intervention group than in the control. The mean score of planning for behavior was significantly decreased in the control group. These findings show that the intervention effectively increased the mean score of participants' planning for behavior. This finding is consistent with a study by Lhakhang *et al*., which showed that the recommended level of planning for action was higher in the experimental group than the control Cwith no intervention).^28^ Moreover, Rashids and Alshehari *Et al*.'s design and planning led to better action taken by nurses to wash their hands.^35^

Another research finding was the significantly higher level of communication skills in nurses in the intervention group than in control. In this research, nurses rated communication skills in a questionnaire. Nurses in the intervention group rated their communication skills higher than their peers in the control group. Through lectures, role-play, and educational movies, promoting communication skills and self-rating was based on an evaluation checklist. Promoting communication skills were found in other studies too. In their research, Devi *et a*l. reported the effectiveness of an intervention based on a video self-rating procedure in improving nursing students' communication skills.^36^ Many studies used multiple interventional methods to promote communication skills. Liaw *et a*l. used a communication skill, self-regulation, problem-solving and emotional support curriculum to promote university students' communication skills.^37^^,^^38^ Their finding was consistent with the present research. The scores for other constructs of the model obtained from the self-rating questionnaire showed the effectiveness of the educational intervention in the experimental group. So far, the existing literature on HAPA has only dealt with the predictors of a specific behavior. The effect of educational interventions has not been reported. 

In the present study, using the intervention based on the HAPA proved to promote nurses' communication skills. Therefore, it is suggested that multiple ways be used to identify barriers to communication and can, thus, help encourage communication skills. An essential point in this research is the nurses' different degrees of interest in rating critical thinking. This difference led to the development of an application for self-rating communication skills and a booklet along with a self-rating evaluation checklist. 

One limitation of this research was the use of self-rating to assess communication skills. In other words, nurses might have overestimated their communication skills. Therefore, a more realistic view can be provided by using different methods to assess communication skills. Moreover, due to the limited sample [to two hospitals], analysis of the HAPA constructs was done only quantitatively. It is suggested that similar interventions be made in larger hospitals with more patients and use the randomization technique to provide more realistic results. Based on recent studies, it is recommended that if communication skills are not at an optimal level in a department or hospital, utilizing educational intervention based on HAPA can guide individuals through communication stages effectively. Additionally, assessing the interventions impact through methods such as 360-degree evaluation is suggested.

Conclusion: The study revealed that HAPA-based intervention can improve nurses' communication skills and can be used as an intervention method.
